# Teachers' Burnout Risk During the COVID-19 Pandemic: Relationships With Socio-Contextual Stress—A Latent Profile Analysis

**DOI:** 10.3389/fpsyt.2022.870098

**Published:** 2022-04-25

**Authors:** Camelia-Mǎdǎlina Rǎducu, Elena Stǎnculescu

**Affiliations:** Department of Psychology, Faculty of Psychology and Educational Sciences, University of Bucharest, Bucharest, Romania

**Keywords:** teacher burnout, work-related stress, psychological profile, COVID-19, online teaching, mixture modeling approach

## Abstract

The purpose of this cross-sectional study was to identify distinct burnout profiles of teachers and to examine their association with work-related stressors, such as workload, students' misbehavior, classroom resources, professional recognition needs and poor colleague relations, as well as socio-demographic variables. Survey data were collected from 330 kindergarten and primary school teachers (84 males, *M*_*age*_ = 38.3, *SD* = 9.14). The latent profile analysis revealed four distinct profiles. The antecedents of teacher burnout (TB) profiles were the stress generated by workload, students' misbehavior, and low professional recognition. The socio-demographic variables, with the exception of gender, were covariates of the TB profiles. The findings implies that career opportunities prospects, classroom management and time-management programs may be useful in preventing teacher burnout.

## Introduction

In this very difficult time for people worldwide, teachers are one of the most challenged groups of workers (1). Being forced to adapt in a short time to new ways of working that include social distancing in classrooms, hybrid teaching and virtual instructions (2), teachers have reported increased levels of anxiety, difficulties in communication and a lack of administrative support (3). All these new stressors proximal to burnout added to the fear generated by COVID-19, which almost all people have experienced (4). A report to UNESCO (5) emphasized the importance of studying the psychological effects of the pandemic on most challenged workers so that the knowledge gained may be applied to prevent and alleviate the difficulties encountered by them in predicted future waves. In this regard, our purpose is to respond to this call by shedding light on stressors that contribute to teacher burnout (TB) in order to help support and enable teachers to meet the next challenges of the pandemic.

## Teachers' Work-Related Stress and Burnout

After decades of research confirming that teaching is a stressful profession (6–8), it has been emphasized that understanding why stress is so pervasive in the field of education can help prevent it (9).

The burnout phenomenon has been conceptualized as a psychological response to prolonged work-related stress that affects one's health and emotional balance (10). The COVID-19 pandemic has affected the social connections of all people, generating new challenges at home and at work (10). In this regard, the socio-contextual burnout proposed by Pietarinen et al. (11) highlights a more social side of teacher burnout and describes three distinct symptoms: (i) exhaustion characterized by a lack of emotional energy and a feeling of being overwhelmed and tired at work; (ii) cynicism represented by detachment from the job in general, as well as from the teaching community and (iii) inadequacy in teacher-pupil interaction. Literature on this particular type of burnout is scarce but very relevant to the current working conditions of teachers.

Extensive previous research on TB has identified several individual and environmental factors that significantly contribute to TB before (12, 13) and during the COVID-19 pandemic (3, 14). Individual factors, such as gender and experience, have been discussed (15, 16). Other individual aspects, such as emotional intelligence (17, 18), personality traits (19, 20) and self-efficacy (14, 21) are also factors that influence TB. In this regard, previous studies have highlighted those seasoned teachers are less likely to experience burnout symptoms compared to younger teachers, while teachers with increased emotional intelligence and self-efficacy but less neurotics are more protected from experiencing burnout symptoms.

Furthermore, the most popular framework that explains the processes involved in professional stress and burnout is Lazarus and Folkman's (22) transactional model. According to this model, the activities undertaken by an individual (cognitive, emotional, behavioral and physiological reactions) to deal with a situation perceived as stressful will or will not allow them to overcome this situation. Additionally, this model emphasizes the importance of the cognitive evaluations that the subject makes of the situation and their own resources (personal and social) and highlights the influence of the individual's attempts to change the situation or themselves through coping strategies. In the educational field, Kyriacou (23) adopted the theoretical conceptualization put forward by Lazarus and Folkman (22) to predict school teachers' reactions. Thus, they defined teacher stress as “the experience by a teacher of negative, unpleasant emotions, such as anger, tension, frustration, depression, which result from a certain aspect of working as a teacher” (23). According to Kyriacou's (24) model of teacher stress, potential stressors are seen as antecedents of teacher stress. The main stressors are physical (e.g., many students in the class) and psychological (e.g., poor colleague relationships). The effect of stressors at work is mediated by coping mechanisms. If coping mechanisms are inadequate, stress occurs. According to the model, teachers' stress is considered to have a negative effect on several dimensions: psychological (e.g., dissatisfaction at work), physiological (e.g., high blood pressure) and behavioral (e.g., absenteeism). Previous studies have examined a wide range of potential variables that have influenced teacher stress, including school environment, classroom and instructional factors (25–28).

Another model introduced to identify precursors of work burnout due to excessive work is the job demands-resources (JD-R) framework (29). In this model, job demands are physical, psychological and social organizational aspects of the job that require physical and/or sustained psychological efforts or skills. Job resources are physical, psychological, social or organizational aspects of the job that are functional in achieving work objectives. These resources are compensatory responses to deficits in meeting demand; they are also recognized as catalysts for growth and development. Stress and burnout result from a subjective mismatch assessed between job demands and resources. A balance of job demands and resources would mean that individuals—in this case, teachers—could successfully fulfill their responsibilities and not experience stress and burnout symptoms. This model has been successfully involved in identifying the resources and job demands that lead to teacher stress and burnout before (30, 31) and during the pandemic (32).

Concerning environmental stressors in the pandemic context, the literature has often discussed organizational factors as important influences on TB. In this regard, time pressure and workload (33, 34), the lack of social and administrative support (9, 35, 36), teaching demands (3) and technostress (37) significantly contribute to TB in the pandemic context. In Romanian settings, before pandemic, as in other countries (19, 31, 38), the work overload and student misbehavior were positively associated with teacher burnout (39), while their low emotional intelligence and high level of neuroticism predicted the onset of burnout symptoms (18). However, the link between stressors and teacher burnout during the pandemic, in a cultural context, has not yet been studied.

In terms of measuring teachers' stressors, Boyle et al. (40) proposed a five-factor model based on teacher stress model (23) that includes workload, students' misbehavior, professional recognition needs, classroom resources and poor colleague relations as main sources of stress for teachers. As far as we know, no previous research has tried to profile TB in relation to stressors in and before a pandemic. Therefore, we examined all dimensions of teachers' stress from Boyle's model and their impact on TB profiles in the pandemic context.

Most of the previous research aimed at profiling TB focused only on clustering it with protective factors, such as self-efficacy, well-being, resilience and coping strategies (16, 41, 42), classroom management and social support (43, 44). Less attention has been paid to identifying those stressors that are the most challenging for teachers and taking into account their predictive role in TB profiles. Therefore, our study goes a step beyond previous studies that identified proactive strategies and other protective factors to emphasize that it is equally important to determine what stressors teachers struggle with during the COVID-19 pandemic.

## Aim of the Study

The main purpose of the current research was to explore how various types of work-related stress among preschool and primary school teachers impact TB risk profiles. More specifically, we first determined whether there were distinct profiles of exhaustion, inadequacy and cynicism that might capture different patterns of TB. Second, we verified antecedents of profiles, namely various types of teacher stress, such as workload, students' misbehavior, professional recognition needs, classroom resources and poor colleague relations. The research design was developed in the framework of the job demands–resources model of burnout (29) and the transactional model of stress proposed by Lazarus and Folkman (22). The job demands–resources model highlights that high workload or work demands and low levels of resources are associated with job strain. According to the transactional model of stress, when one's perceived ability to cope is exceeded by perceived demands, the stress response intensifies.

Based on previous studies that highlighted burnout symptoms experienced by teachers in the COVID-19 pandemic (3, 16, 42), we determined the first research question (RQ):

(RQ1) Are there different teacher profiles in terms of experienced socio-contextual burnout consisting of exhaustion, cynicism toward the professional community and inadequacy in teacher –pupil interaction during the COVID-19 pandemic?

Furthermore, considering previous studies on the relationship between stressors such as workload (34), professional recognition (8, 45), student misbehavior (38), classroom resources (19), social support (44) and TB, we developed the next question:

(RQ2) Do teachers with the different profiles differ from each other in terms of experienced stressors, such as workload, professional recognition needs, students' misbehavior, classroom resources and poor colleague relations, during the COVID-19 pandemic?

Taking into account the previous study that highlighted the association between socio-demographic variables and TB profiles (16), we determined the last question:

(RQ3) Are socio-demographic variables—that is, gender, teaching level, professional experience and urban or rural teaching environment—covariates of TB profiles?

## Methods

### Participants

Our sample included 330 educators (75% women, *M*_*age*_ = 38.3 years, *SD* = 9.14), of which 108 worked at the preschool level and 222 at the primary school level. Their reported professional experience was less than two years (4.5%), between two and five years (10.9%), between five and 10 years (19.1%), between 10 and 20 years (25.5%) and more than 20 years (40%). Using a convenience sampling method, we selected the teachers from the register of district Teachers Council The total response rate of the e-mail paper survey sent to teachers was 45%. The selection criteria for inclusion in this study were a primary or preschool level of teaching.

### Procedure

The current study had a cross-sectional design based on responses to a survey that comprised three sections. The first section included the study details, the informed consent and the guaranteed confidentiality of all data obtained. The second section included participants' socio-demographic information, such as gender, teaching grades, years of professional experience and urban or rural teaching environment. The last section involved reporting the levels of burnout and stress. The study was conducted in accordance with the Declaration of Helsinki and the recommendations and approval by Bucharest University Ethics Committee (no 11/26.04.2021). Data were collected during the 2021 spring break via Google Forms, the questionnaires being sent to teachers by e-mail.

### Measures

The Romanian translation of all measures used in the current study was performed according to the recommended forward-backward translation procedures described by Sousa and Rojjanasrirat (46).

The Socio-Contextual Teacher Burnout Inventory (STBI) (11) was used to measure TB. This nine-item scale (sample item: “*With this work pace, I don't think I'll make it to the retiring age”*) employed a Likert scale from 1—completely disagree to 7—completely agree. The established three constructs were teacher exhaustion *(item e.g., “I feel burnt out.”)*, cynicism toward the teacher community *(item e.g., “I often feel like an outsider in my work community.”)* and inadequacy in the pupil–teacher relationship *(item e.g., The challenging pupils make me question my abilities as a teacher.”)*. There is currently no study in the literature that indicates a cut-off for this scale. Therefore, according to the study of Pyhältö et al. (16), we considered that as the scores are higher, the burnout level is also higher. More precisely, we considered that depending on the answers given on the Likert scale from 1 to 7, we will have the following thresholds: 1–3—no burnout; 4–6—very low burnout; 7–9—mild burnout; 10–12—moderate burnout; 13–15—high moderate burnout; 16–18—high burnout; 19–21—very high burnout. In our sample, STBI proved very good psychometric properties in terms of: (i) internal consistency (ω_*hierarchical*_ = 0.91, ω_*exhaustionl*_ = 0.88, ω_*inadequacy*_ = 0.85, ω_*cynicism*_ = 0.80; *CR* = 0.94); (ii) convergent validity (AVE = 0.64); and (iii) construct validity (CFI = 0.97, TLI = 0.95, RMSEA = 0.06, CI [0.04, 0.09], SRMSEA = 0.02, λ_*s*_ ranged between 0.57 and 0.86).

The Teacher Stress Inventory (TSI) measures work-related teacher stress (40). This scale comprises 20 items (e.g., “*Level of stress concerning noisy pupils”*) and uses a Likert scale from 0—*no stress* to 4—*extreme stress* to assess teachers' stress in five dimensions—workload *(item e.g., “Level of stress concerning to much work to do”*), students' misbehavior *(item e.g., “Level of stress concerning maintaining class discipline.”*), professional recognition needs *(item e.g., “Level of stress concerning poor career structure/poor promotion prospects.”)*, classroom resources *(item e.g., “Level of stress concerning lack of time to spend with individual pupils.”)* and poor colleague relations *(item e.g., “Level of stress concerning attitudes and behaviors of other teachers.”)*. In our sample, TSI proved good psychometric properties in terms of: (i) internal consistency (ω_*hierarchical*_ = 0.92, ω_*profrecognition*_ = 0.80, ω_*stdbehavior*_ = 0.88, ω_*workvol*_ = 0.60, ω_*workresources*_ = 0.87, ω_*relations*_ = 0.82; *CR* = 0.94); (ii) convergent validity (AVE = 0.53); and (iii) construct validity (CFI = 0.95, TLI = 0.93, RMSEA = 0.07, CI [0.05, 0.09], SRMSEA = 0.03, λ_*s*_ ranged between 0.59 and 0.83). Socio-demographic variables such as gender, teaching level, professional experience and urban or rural teaching environment were collected.

### Data Analysis

Latent profile analysis (LPA) was performed to identify sets of mutually exclusive and exhaustive latent profiles using continuous indicator variables, that is, the three dimensions of TB—exhaustion, inadequacy and cynicism. LPA is a mixture modeling technique by which groups of people are captured based on similarities in their responses to various research variables, in our study the three dimensions of TB. LPA analysis was conducted using Mplus 8.6 software (47). The robust maximum likelihood (RML) estimator was used, as it produces robust standard errors to handle non-normally distributed data. Models with 2–5 classes were considered, each with three indicators, that is, the dimensions of TB. We run Monte Carlo analysis to compute the specific fit indicators for statistical power to detect the correct number of profiles in LPA, as recommended by Tein et al. (48) and Spurk et al. (49). Optimal model selection was based on several information criteria—log likelihood (LL), Akaike information criterion (AIC), Bayesian information criterion (BIC), sample size-adjusted BIC (SSA-BIC) and entropy R^2^. Lower values for the AIC, BIC and SSA-BIC indicate a better balance between model fit and parsimony, while higher values for entropy (i.e. > 0.80) indicate better classification utility and class separation. Supplementary tests—an adjusted Lu-Mendell-Rubin likelihood ratio test (aLMR) and a bootstrap likelihood ratio test (BLRT)—were performed in order to compare the subsequent models. A statistically significant test result (*p* < 0.05) indicates that the model with k classes fits the data better than the model with one latent class less, that is, k-1 classes (50). Additionally, solution stability was checked to assure the maximum likelihood solution is replicated using multiple sets of random starting variables. Model identification was evaluated with 1,000 sets of random starting values for all models, 100 iterations and 100 solutions retained for final stage optimization. After identification of the profiles, we testified the predictive role of various types of teacher stress on profile membership using multinomial logistic regression computed with the R3STEP procedure. Baseline-category multinomial logistic regression provides the increase in odds of membership in a target latent profile compared to other profiles for one-unit increases in the predictor, that is, various types of teacher stress. The association between socio-demographic variables—gender, teaching level, professional experience and urban/rural teaching environment—and profile membership was conducted based on multinomial logistic regression with an R3STEP approach.

## Results

The descriptive statistics for sociodemographic variables are shown in Table 1.

**Table 1:** Descriptive statistics for sociodemographic variables.

**Variable**	**Frequency (Valid%) or Mean (** ***SD*** **)**	
Sociodemographic
Gender	Male	84 (25.5%)
	Female	246 (74.5%)
Teaching level	Preschool	108 (32.7%)
	Primary school	222 (67.3%)
Professional experience(years)	<2	15 (4.5%)
	2–5 36	(10.9%)
	6–10	63 (19.1%)
	11–20	84 (25.5%)
	>20	132 (40.0%)
Urban/rural environment	Urban	217 (65.8%)
	Rural	113 (34,2%)

Furthermore, the profile indicators and predictors of profile membership are depicted in Table 2.

**Table 2:** Profile indicators and predictors of profile membership.

	***M* (*SD*)**	**Min**	**Max**	**Skewness**	**Kurtosis**
Exhaustion	12.18 (4.51)	3	21	−0.10	−0.80
Inadequacy	9.79 (4.88)	3	21	0.36	−0.90
Cynicism	11.66 (5.31)	3	21	−0.19	−1.24
Stress workload	4.38 (1.99)	0	8	−0.47	−0.40
Stress students' misbehavior	11.64 (5.87)	0	24	−0.04	−0.94
Stress low professional recognition	3.71 (2.06)	0	8	0.04	−0.59
Stress low resources	4.76 (2.41)	0	8	−0.20	−1.06
Stress poor colleagues relations	5.06 (3.05)	0	12	0.21	−0.85

### Latent Profile Solutions

Fit statistics from the LPA models, that is, two-profile to five-profile solutions, are set out in Table 3. As can be seen, gradual improvement was observed up to the four-profile solution, and the five-profile solution decreased the quality of the classification. the significant V-L-M-R Likelihood results (<0.5) averaged over replications as indicating that the study had enough power or capacity to correctly recover a four-profile vs. a three-profile solution Although some of the fit indicators—LL, AIC, BIC and SSQ-BIC—had the lowest values for the five-profile solution, entropy was lowest and the best loglikelihood value has been not replicated for the model including five-profile solution. The aLMR value was not significant for the five-profile solution but was significant for the four-profile solution. Consequently, these results lent support for the four-profile solution as the best fitting model for the present study's data. Additionally, in the four-profile model, the average latent profile probabilities for the most likely profile were 0.97, 0.87, 0.94 and 0.91. All were well above the cut-off (> 0.80) recommended by Watson et al. (51).

**Table 3:** Model fit information for latent profile analysis.

**No. of profiles**	**Free parameters**	**LL**	**AIC**	**BIC**	**SSA-BIC**	**Entropy**	**aLMR**	**BLRT**
2	10	−2704.76	5429.52	5467.51	5435.79	0.880	518.40 (0.00)	0.00
3	14	−2588.96	5205.92	5259.10	5214.69	0.897	222.03 (0.00)	0.00
**4**	**18**	–**2561.22**	**5158.45**	**5226.83**	**5169.74**	**0.883**	**53.17 (0.00)**	**0.00**
5	22	−2531.01	5105.03	5189.61	5119.83	0.879	57.92 (0.15)	0.00

*Bold indicates best fitted model. LL, log likelihood; AIC, Akaike information criterion; BIC, Bayesian information criterion; SSA-BIC, sample size adjusted BIC; aLMR, adjusted Lu-Mendell-Rubin likelihood ratio test; BLRT, bootstrapped likelihood ratio test*.

### Four-Profile Model of TB Risk

**Figure 1:**
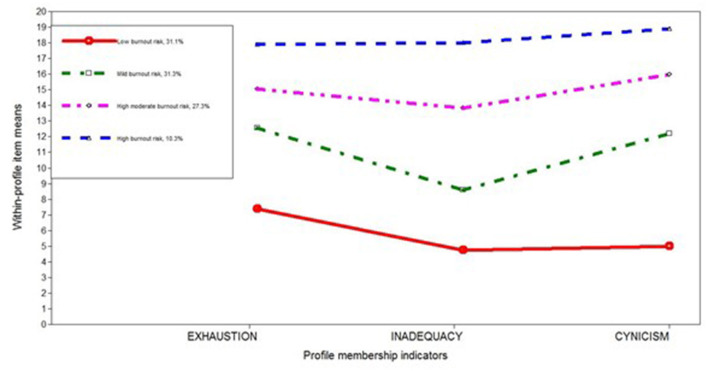
Parameter estimates for the four-profile model of TB risk; within-profile item means.

The model best fitted to our data, the four-profile model of TB risk, is depicted in Figure 1, taking into account within-profile item means obtained for each indicator of profile membership, that is exhaustion, inadequacy and cynicism.

Parameter estimates for overall item respectively within-profile item means for the four-profile model are set out in Table 4. As can be seen, the first profile, *Low burnout risk*, included 31.2% of the teachers and was characterized by low levels of exhaustion and very low levels of inadequacy in the teacher–student relationship and cynicism toward the professional community. These results are significantly less than those obtained in all other profiles. The second profile, *Mild burnout risk*, included 31.2% of the teachers and was defined by moderate levels of exhaustion and cynicism on the one hand and low levels of inadequacy on the other. Therefore, the teachers with this profile have mild burnout risk, especially in terms of exhaustion and cynicism. The third profile, *High moderate burnout risk*, included 27.3% of the teachers and was characterized by high moderate exhaustion, inadequacy and cynicism. These results reveal a simultaneously increased pattern of all three symptoms of TB risk. The final profile, *High burnout risk*, included 10.3% of the teachers and was characterized by a similar pattern to the previous one but with higher levels of all three indicators.

**Table 4:** Parameter estimates for the four-profile model.

		**Low risk**	**Mild risk**	**High moderate risk**	**High risk**
		**1**	**2**	**3**	**4**
**(l0ptr0pt)3-6 Profile prevalence**		***n* = 103**	***n* = 103**	***n* = 90**	***n* = 34**
**Profile indicators**	**Overall item means**	**Within-profile means**			
	(***SD***)	**Estimate** (***SE***)			
Exhaustion	12.18 (5.31)	7.39 (0.29)	12.56 (0.32)	15.04 (0.36)	17.90 (0.79)
Inadequacy	9.79 (4.88)	4.74 (0.18)	8.59 (0.22)	13.82 (0.46)	18.02 (0.59)
Cynicism	11.69 (4.51)	4.97 (0.17)	12.18 (0.33)	15.96 (0.23)	18.87 (0.54)

### Antecedents of Latent Profiles

Having as reference the *Low burnout risk* profile, we noticed there was a significant tendency to increase the sources of stress generated by workload and students' behavior but only at the level of the *Mild burnout risk* and *High moderate burnout risk* profiles. In other words, these types of stress have a more pronounced impact on the high moderate than the mild burnout risk profile. Our findings revealed an interesting pattern. In the case of the *High burnout risk* profile, although the odds ratio (OR) for stress generated by workload was > 1, it did not reach the threshold of statistical significance and did not have a significantly higher contribution to this profile membership. The same pattern was obtained in the case of stress related to poor colleagues relations, that is OR > 1, *p* = 0.64. The stress generated by students' misbehavior did not have a significant impact either (OR < 1, *p* > 0.05). Furthermore, the results showed that the only significant contribution was the stress generated by low professional recognition, as set out in Table 5.

**Table 5:** Effects of predictors on membership in latent profiles of TB risk. Odds ratios (OR), 95% confidence interval for the effects of work-related stress on TB profile membership.

**Burnout profile**	**Odds ratio (OR)**	**LL2.5%**	**UL2.5%**
Reference profile: Low burnout risk
“**Mild burnout risk**”
Stress workload	2.190^***^	1.399	3.429
Stress student's misbehavior	1.410^***^	1.241	1.601
Stress professional recognition	1.180	0.911	1.528
Stress classroom resources	0.669	0.518	0.864
Stress poor relations	1.218	0.944	1.573
“**High moderate burnout risk**”
Stress workload	3.139^***^	1.878	5.248
Stress student's misbehavior	2.146^***^	1.761	2.615
Stress professional recognition	0.971	0.677	1.392
Stress classroom resources	0.532	0.374	0.756
Stress poor relations	1.197	0.840	1.706
**High burnout risk**
Stress workload	1.314	0.778	1.927
Stress student's misbehavior	0.865	0.387	1.937
Stress professional recognition	5.664^**^	3.124	8.178
Stress classroom resources	0.351	0.081	1.515
Stress poor relations	1.109	0.902	1.986

*LL, lower limit of the confidence interval; UL, upper limit*.

** *p < 0.01*.

*** *p < 0.001*.

### Socio-Demographic Variables as Covariates of TB Profile Membership

The results proved that gender did not have a significant impact on TB profiles. On the contrary, all the other socio-demographic variables were significant predictors of profile membership. In terms of teaching level, preschool teachers had higher odds (*OR* = 1.42, 95% CI [1.12, 1.81]) of belonging to the *Mild burnout risk* profile than to the *Low burnout risk* profile. Furthermore, our findings show that teachers with high professional experience had higher odds (*OR* = 6.20, 95% CI [2.79, 13.81]) of belonging to the *High burnout risk* profile than to the *Low burnout risk* profile. Comparing teachers according to urban/rural teaching environment, we found that those from rural schools had higher odds (*OR* = 2.11, 95% CI [1.77, 2.50]) of belonging to the *Higher moderate burnout risk* profile than to the *Low burnout risk* profile.

## Discussion

Our findings show that TB profiles were classified into four different categories: *Low burnout risk, Mild burnout risk, High moderate burnout risk* and *High burnout risk*. The profiles differed in all three symptoms of burnout—exhaustion, cynicism toward the teacher community and inadequacy in the pupil–teacher relationship. In addition, differences were found concerning teachers' stressors between profiles in terms of various types of stress, more precisely workload, students' misbehavior and professional recognition needs.

The first latent profile from our analysis was *Low burnout risk*. The teachers belonging to this profile displayed low levels of exhaustion, very low levels of inadequacy in the teacher–pupil relationship and cynicism toward the professional community. Additionally, the findings revealed that the levels of all three indicators of profile membership were statistically significantly lower than those in the other profiles. It is not surprising that teachers with the *Low risk burnout* profile do not experience symptoms of inadequacy in interaction with pupils and cynicism because previous studies have already highlighted that teachers who are less stressed are more efficacious (15, 21) and have better relationships with pupils (52, 53).

The second profile, *Mild burnout risk*, was characterized by moderate levels of exhaustion and cynicism toward the professional community on one side and low levels of inadequacy on the other side. Our findings proved that these mild burnout symptoms seem to be generated by the increase in stress generated by workload and students' misbehavior. Along the same lines, moderate exhaustion and cynicism was identified among Canadian teachers (32), where exhaustion was correlated with job demands.

The third profile, *High moderate burnout risk*, was characterized by teachers with high moderate symptoms of exhaustion, inadequacy in interactions with pupils and cynicism toward the professional community. In this case, the burnout symptoms are fueled by workload and students' misbehavior but with a stronger impact than in the case of teachers with the *Mild burnout risk* profile. In this regard, it seems that even during the pandemic period, workload and students' misbehavior remained the main stressors as before the pandemic (19, 24, 38).

The *High burnout risk* profile, the profile with the lowest prevalence (*n* = 34), represented the teachers who experienced higher levels of exhaustion, cynicism toward the teaching community and inadequacy in teacher–pupil interaction. In examining the *High risk burnout* profile, we noticed the teachers within it registered a high level of cynicism toward the professional community, unlike those in a study of Finnish teachers (16). One explanation could be that in our sample the largest source of stress that had a statistically significant difference from other profiles was that generated by low professional recognition. If the feeling of inadequacy might be more closely related to intra-individual issues, such as self-esteem and general self-efficacy (41, 54), cynicism toward the professional community reflects dissatisfaction that has a rather external source than a dispositional trait (26, 55, 56) revealed correlates of cynicism such as social support, organizational commitment, and work–family or family–work conflict. It seems plausible that online teaching during the COVID-19 pandemic increased the level of stressors encountered by teachers, which in turn could affect organizational commitment and affective engagement and could accentuate possible previous family–work conflicts.

Another explanation could be related to the fact that professional recognition needs are translated into remuneration, career promotion prospects and social recognition (24, 40). As it is already recognized in JD-R model (29) that the lack of resources compared to demands would result in stress, which might eventually lead to TB and attrition (28) and that the perceived imbalance of effort and reward is associated with a high risk of developing burnout symptoms (57), it seems that for teachers with this profile the lack of gratification increased their burnout symptoms. This increase in the need for professional recognition could be due to the fact, as previous studies conducted during the pandemic have shown (10, 37, 58), that new teaching conditions produced new stressors for teachers and forced them to put extra effort into the teaching process (59), and thus the need for reward increased. Furthermore, despite that fact that stressors such as students' misbehavior and workload were present among teachers with the *High-risk burnout* profile, they did not make a significantly higher contribution for this profile membership compared to both previous ones.

Navigating through profiles from the high-risk level to the low risk level we gain valuable understanding of TB. In our case, the increased stress and frustration related to the lack of professional recognition in terms of remuneration, social recognition, and career opportunities together with the increased level of cynicism are what burdens teachers in this profile the most. Moreover, teachers' stress related to financial compensation was also identified among Bulgarian teachers (60), Slovak teachers (61), Greek teachers (62) and Turkish teachers (63) in the pandemic period. Thus, these challenging times seem to accentuate even more teachers' frustration related to professional recognition, especially in some European countries. Accordingly, further studies, particularly longitudinal person-oriented studies on professional recognition needs, are needed to test these assumptions.

As Huttell et al. (64) explained, burnout is not stable in nature and profile grouping is not a stable individual trait. The progression toward burnout and profile grouping can be reversed based on changes in the relationships between resources and demands. In this regard, burnout symptoms can appear anytime and have increased alarmingly during the COVID-19 pandemic, thus putting teachers' mental health at risk.

In the case of the average burnout risk profiles *Mild burnout risk* and *Moderate burnout risk*, differences from the reference profile, *Low burnout risk*, were found in terms of stress related to workload and students' misbehavior. Interestingly, the teachers belonging to this profile, even though they live under the same contextual settings, were not affected by the lack of social/professional recognition. Rather, they were stressed only by workload and students' misbehavior. One explanation for the fact that teachers belonging to these profiles were not affected by the lack of social/professional recognition could be that the frustrations related to lack of professional recognition were absorbed by their interest in the quality of their instructional process (7). Thus, according to the JD-R model (29), their passion and vocation were important internal resources that offset the new demands of the workplace, but they experienced limited resources in managing the increased workload generated by the new teaching conditions and a lack of resources in managing virtual relationships with students, being unprepared to find ways to maintain discipline in the online classroom (32).

The present study makes several contributions to the literature on TB (11, 16). First, it expands the body of research on TB in the challenging context of online teaching related to the COVID-19 pandemic. Second, it adds to the few studies that have been based on the mixture modeling approach, more specifically LPA, and has the advantage of having a person-centered approach rather than a variable-centered approach. Third, it is the first study to our knowledge that analyses the various types of work-related stress as antecedents of TB profiles. More precisely, the high levels of stress related to students' misbehavior and workload were related to a high probability of belonging to the *Mild burnout risk* and *High moderate burnout risk* profiles, while the high level of stress related to professional recognition needs generated a high probability of belonging to the *High burnout risk* profile. In this regard, it seems that the pandemic period accented stressors such as workload and students' misbehavior, which were reported even before the pandemic (24, 27, 38) as major inconveniences for teachers. To these was added a new stressor proximal to burnout, the lack of professional recognition.

In terms of socio-demographic variables, our findings revealed no association between gender and profile membership, which is in line with Pyhältö et al.'s (16) study. Preschool teachers had higher odds than primary school teachers of being included in the *Mild burnout risk* profile than in the *Low burnout risk* profile. One explanation for this finding could be the fact that the pandemic brought up new routines, such as extra handwashing, stricter sanitation requirements, different teacher–child ratios, prohibiting parents from entering preschools and social distancing (65, 66), all of which increased kindergarten teachers' physical and mental stress. As mentioned, an interesting pattern emerged; in our research, teachers with higher professional experience had a higher burnout risk in the context of remote learning related to the COVID-19 pandemic than less experienced teachers. However, it seems plausible if we take into account that in online teaching younger teachers have been more advantaged due to stronger digital skills compared to older teachers who have encountered greater difficulties in adapting to e-learning systems (67, 68). Additionally, teachers in rural educational environments were at high moderate risk of burnout compared to those teaching in urban environments. This finding seems to be very relevant, first because the lack of infrastructure for broadband access in some rural areas often constrained rural teachers in terms of having to operate with fewer resources than their urban counterparts (69).

The current study has some limitations that should be not-ed. First, the study used a convenience sampling technique; therefore, the findings cannot be generalized. Second, the use of cross-sectional data in this study necessitates further longitudinal and experimental design studies. More specifically, the cross-sectional design and R3STEP approach used in the specified mixture model reveal various exogenous covariates of burnout profiles, that is, work-related stressors but no causal links with TB. Therefore, future longitudinal studies are needed to capture how to evolve the relationship between various types of work-related stress and socio-contextual burnout after the remote learning period and after the COVID-19 pandemic. In addition, in terms of future research directions, more studies are needed to find the factors that lead to increased levels of cynicism toward professional community and explore if they are either intra-individual or socio-contextual in nature. Additionally, because a broader cul-tural context is very complex but was not the focus of the present article, further cross-cultural comparative studies on burnout should be conducted.

### Practical Implications

Considering the prevalence of teachers in two of four profiles determined in the present study, namely *High moderate risk* and *High burnout risk*, it should be considered a priority to identify as early as possible the teachers at risk for the onset of burnout symptoms and those who have difficulty managing work-related stress. Taking into account that the most common antecedents or risk factors for TB proved to be workload and students' misbehavior, it is tremendously important that teachers at risk ask for and receive help so that the burnout symptoms do not affect their health, their relationships with pupils and their personal lives. In this regard, the closest resource is social support (53). For example, collaborative teaching could help in buffering burnout symptoms by sharing work, asking for advice, bringing out concerns and receiving help from co-workers (70). In addition, an intervention program based on cognitive behavioral therapy and mindfulness-based stress reduction has proven effective in reducing TB (71) and should be considered for implementation in order to support teachers' mental health during the pandemic and beyond.

Concerning professional recognition needs that proved to be a major stressor, we highlight that teachers may be able to tolerate a greater workload if they feel they are well-rewarded for their efforts and if they value their work with children (72). Therefore, our study encourages cross-cultural learning and sharing among preschool and primary school teachers through teacher exchanges and collaborations that can generate a unique understanding of the best ways to fight TB.

## Conclusions

In summary, our study expands the empirical body of research on TB risk (16, 20, 32, 53) by being the first study to explore TB symptoms and work-related stressors during the COVID-19 pandemic using a person-centered approach. The results showed that over half of the teachers in our sample were affected to varying degrees by low to high burnout symptoms. Four TB profiles were identified. Workload, students' misbehavior and the lack of professional recognition were the major stressors that contribute the most to TB profile membership. The pandemic context brought to light a new stressor proximal to TB—professional recognition needs. In this regard, the present study highlights that educational managers could support teachers' health and well-being by: (1) knowing teachers' needs, worries and stressors in order to prevent the appearance of symptoms of exhaustion, inadequacy in the teacher–pupil relationship and cynicism toward the professional community; (2) decreasing burnout through supportive programs based on developing skills for classroom management in various learning environments, time management and work–life balance; and (3) professional development programs that promote career opportunities such as self-actualization, visibility and social recognition.

## Data Availability Statement

The raw data supporting the conclusions of this article will be made available by the authors, without undue reservation.

## Ethics Statement

The studies involving human participants were reviewed and approved by University of Bucharest Ethics Committee (no 11/26.04.2021). The patients/participants provided their written informed consent to participate in this study.

## Author Contributions

C-MR contributed in conceptualization, developed the questionnaires, and collected the data. ES and C-MR developed the analytical plan, undertook the statistical analyses, interpreted the results of the statistical analyses, and wrote the paper. Both authors have read and agreed to the published version of the manuscript.

## Conflict of Interest

The authors declare that the research was conducted in the absence of any commercial or financial relationships that could be construed as a potential conflict of interest.

## Publisher's Note

All claims expressed in this article are solely those of the authors and do not necessarily represent those of their affiliated organizations, or those of the publisher, the editors and the reviewers. Any product that may be evaluated in this article, or claim that may be made by its manufacturer, is not guaranteed or endorsed by the publisher.
